# Periodic heat waves-induced neuronal etiology in the elderly is mediated by gut-liver-brain axis: a transcriptome profiling approach

**DOI:** 10.1038/s41598-024-60664-9

**Published:** 2024-05-08

**Authors:** Subhajit Roy, Punnag Saha, Dipro Bose, Ayushi Trivedi, Madhura More, Christina Lin, Jie Wu, Melanie Oakes, Saurabh Chatterjee

**Affiliations:** 1 Environmental Health and Disease Laboratory, Department of Environmental and Occupational Health, Program in Public Health, Susan and Henry Samueli College of Health Sciences, University of California, Irvine, CA 92697 USA; 2 Genomics Research and Technology Hub, Department of Biological Chemistry, School of Medicine, University of California, Irvine, CA 92697 USA; 3 Division of Infectious Diseases, School of Medicine, University of California, Irvine, CA 92697 USA; 4 Long Beach VA Medical Center, Long Beach, CA 90822 USA

**Keywords:** Hyperthermia, Human health, Gut-liver-brain axis, ORM2, RANTES, Climate change, Microbiota, Molecular medicine

## Abstract

Heat stress exposure in intermittent heat waves and subsequent exposure during war theaters pose a clinical challenge that can lead to multi-organ dysfunction and long-term complications in the elderly. Using an aged mouse model and high-throughput sequencing, this study investigated the molecular dynamics of the liver-brain connection during heat stress exposure. Distinctive gene expression patterns induced by periodic heat stress emerged in both brain and liver tissues. An altered transcriptome profile showed heat stress-induced altered acute phase response pathways, causing neural, hepatic, and systemic inflammation and impaired synaptic plasticity. Results also demonstrated that proinflammatory molecules such as S100B, IL-17, IL-33, and neurological disease signaling pathways were upregulated, while protective pathways like aryl hydrocarbon receptor signaling were downregulated. In parallel, Rantes, IRF7, NOD1/2, TREM1, and hepatic injury signaling pathways were upregulated. Furthermore, current research identified Orosomucoid 2 (ORM2) in the liver as one of the mediators of the liver-brain axis due to heat exposure. In conclusion, the transcriptome profiling in elderly heat-stressed mice revealed a coordinated network of liver-brain axis pathways with increased hepatic ORM2 secretion, possibly due to gut inflammation and dysbiosis. The above secretion of ORM2 may impact the brain through a leaky blood–brain barrier, thus emphasizing intricate multi-organ crosstalk.

## Introduction

In a dynamically changing global landscape, the imminent threat of climate change is evident in rising temperatures, raising concerns about intermittent heat waves leading to acute and chronic heat stress, where the aging population is especially vulnerable^1^. Heat stress due to the deployment of troops in the Southwest Asian region and during combat training in arid regions also have a deleterious effect on the health of aging Veterans. The Southwest Asia regional climate is hot and arid, which exposes military personnel to intense heat and sunlight during the day, posing long-term health consequences for veterans^2^. Heat stress, a heat-related illness, occurs when the body's cooling mechanisms fail, leading to a rapid increase in the body temperature^3^ and cause pathophysiological changes and fatalities, with excess mortality linked to summertime temperatures surpassing long-term averages and heat extremes due to heatwaves^4^. Recent CDC data reports over three thousand deaths in about two years solely due to heat exposure, indicating a gradual increase in heat stress-related deaths with age, peaking among those aged fifty-five to sixty-four^5^. Various factors, including housing conditions, limited mobility, financial constraints, psychological issues, ignorance, and comorbid conditions, heighten the elderly's susceptibility to heat stress^6^. Alarming predictions estimate over a ninety percent increase in heat stress-associated deaths in New York by 2050 and up to a seven-fold increase in California by 2090^7,8^.

Heat stress intricately affects vital organ systems, particularly the cerebral and hepatic domains^9^. In the neurophysiological landscape, heat stress induces cellular perturbations, leading to cerebral edema^10^. Experimental animal models showed acute heat stress activating astrocytic processes, causing neuronal damage in the cerebral cortex^11^. Simultaneously, the neuroinflammatory environment, marked by microglial activation, pro-inflammatory cytokine release, and blood–brain barrier (BBB) disruption, contributes to cognitive decline, compromises adult neurogenesis, and age-related pathologies, significantly impacting the geriatric population^12,13^. The liver, an indispensable organ of metabolic orchestration, maintains pivotal processes encompassing ammonia purification, biosynthesis of vitamins and minerals, energy homeostasis, and the regulation of fundamental physiological equilibrium^14^. Intriguingly, the liver emerges as a remarkably sensitive organ to the toxic effects of heat stress. Acute thermal stress instigates metabolic perturbations in the liver via oxidative stress^15^. In a chronic heat stress milieu, the liver succumbs to apoptosis triggered by endoplasmic reticulum (ER) stress, concomitant impairment in ammonium detoxification, elevation of plasma-ammonia levels, and aggravated hepatic pathologies^16^. Additionally, heat stress was known to promote dysfunction in hepatic autophagy, thereby unveiling its multifaceted capacity to inflict hepatic damage^17^. To date, no studies have investigated the integrative effects of heat stress on the liver and brain axis beyond specific organ-level pathologies and molecular intricacies. Therefore, this study aimed to uncover the molecular interplays of the liver-brain connection in heat stress-related pathologies together with individual organ-specific pathological changes using transcriptome approaches.

High-throughput transcriptomic approaches play a pivotal role in understanding RNA expression changes under specific disease and stress conditions. Transcriptome profiling has been one of the most used techniques for molecular investigations of human diseases, which has aided the discovery of several molecular biomarkers and possible therapeutic targets for a range of disease pathologies. In cancer biology, transcriptome profiling has been instrumental in identifying prognostic and predictive biomarkers crucial for personalized medicine development^18^. It has also been employed to reveal blood transcriptomic signatures associated with molecular changes in the brain and clinical outcomes in neurological disease^19^. One study used brain endothelial transcriptome profiling to highlight persistent post-restoration changes in gene expression that lead to disruption of the blood–brain barrier (BBB) in various neurological diseases, thus offering potential targets for the treatment^20^. Past studies discussed the impact of RNA sequencing on understanding complex diseases, including neuropsychiatric disorders, through analysis of mRNA from transgenic models and human tissues^21^. Formerly, transcriptomic profiling of cardiac and skeletal muscle was applied to study heat stress responses in chickens adapted to different altitudes^22^. Cattle liver and intestinal transcriptomics identified heat stress-related genes and pathways^23–25^. *Caenorhabditis elegans* displayed global gene expression shifts under heat stress^26^. Bovine granulosa cells showed transcriptomic changes in redox, inflammatory, and metabolic pathways during acute heat stress^27^. Human studies revealed rapid gene expression changes in blood cells during extreme heat exposure, impacting stress signaling, metabolism, and immune response^28^. These studies underscore transcriptomics' utility in elucidating molecular mechanisms in response to heat stress across species. A research gap is evident, prompting a multiorgan transcriptomic study in genetically more identical animal models to humans such as laboratory mice with a 90% genomic similarity to humans^29^. Notably, no functional multiorgan transcriptomic study was conducted on heat stress effects in the aging brain and liver, where age is a known potential risk factor for related disease pathologies and mortality. Hence, this study focused on delving into the transcriptional profiling of the liver, brain, and liver-brain axis in an aging mouse model exposed to heat stress, exploring the molecular interplays behind the manifested pathophysiology, and correlating that through tools of bioinformatics, systems biology followed by functional validations.

## Results

### Periodic heat stress showed distinctive gene expression patterns in brain and liver tissues of aging mice

**Figure 1:**
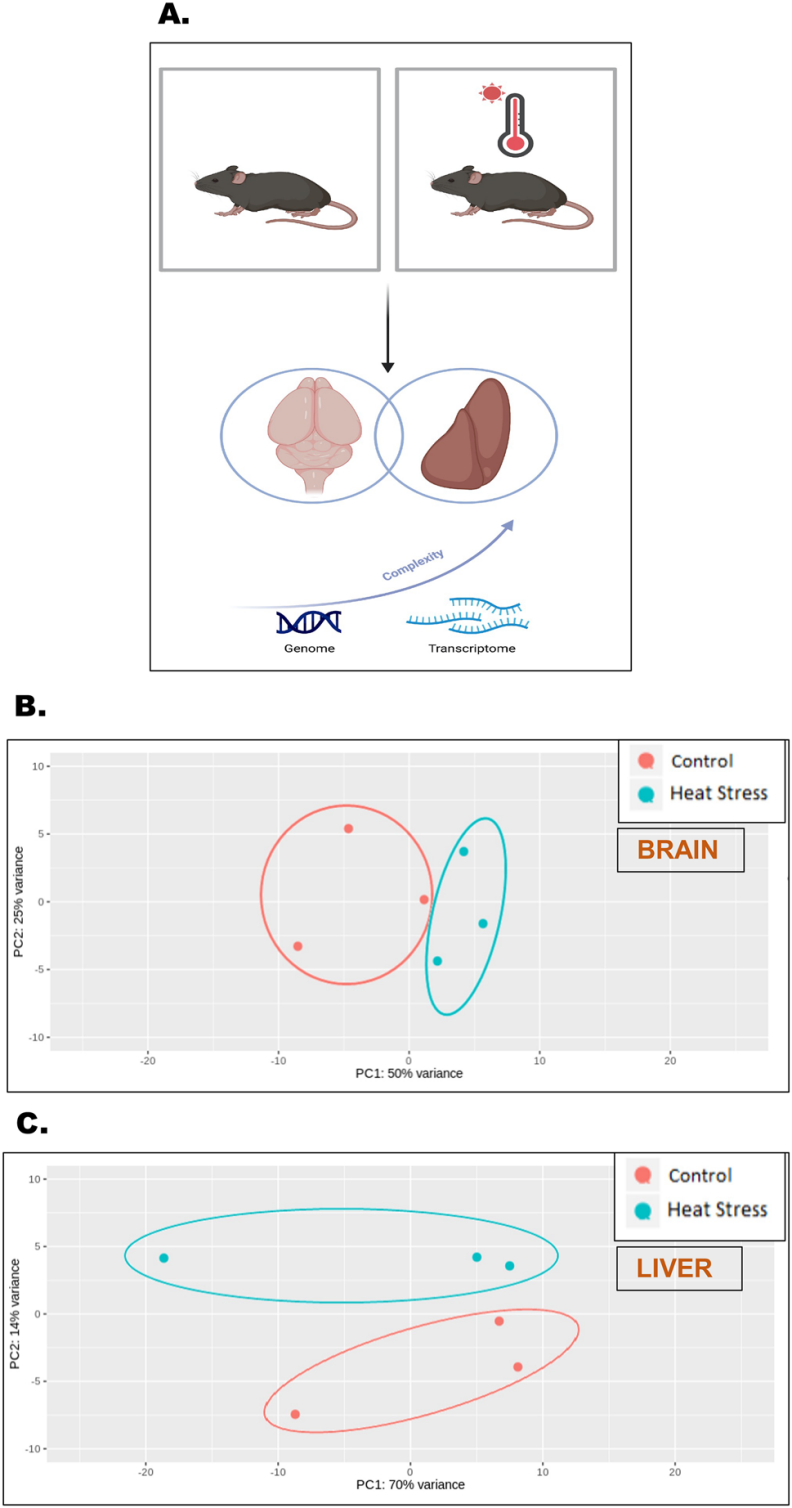
Periodic heat stress showed distinctive gene expression patterns in brain and liver tissues of aging mice. (**A**) Schematic representation of the basic experimental design. (**B**) Principal component analysis plot of brain tissues comparing the differential gene expressions between the control (red) and heat stress (blue) groups, Principal component 1 (PC1) and 2 (PC2) plotted along abscissa and ordinate respectively (*PC1 at 25% and PC2 at 50% variance*). (**C**) Principal component analysis plot of brain tissues comparing the differential gene expressions between the control (red) and heat stress (blue) groups, Principal component 1 (PC1) and 2 (PC2) plotted along abscissa and ordinate respectively (significance was set conventionally with *PC1 at 25% and PC2 at 50% variance*).

Periodic heat stress was induced in an aging mouse model (C57BL6/J wild type) of 24 months at 40 ± 0.5 °C with a relative humidity of 60% ± 5% for three hours/day for 15 days. After euthanization, brain and liver tissues were harvested and processed for transcriptomic evaluation (Fig. 1A). The differential gene expression patterns revealed by transcriptomics showed significantly distinct changes in gene expression profiles in both brain and liver tissues were plotted through principal component analysis when compared between control and heat stress groups (Fig. 1B,C, *PC1 at 25% and PC2 at 50% variance*).

### Heat stress-induced transcriptional alterations in both the brain and liver entailed discernible shifts in the expression patterns of pivotal genes

**Figure 2:**
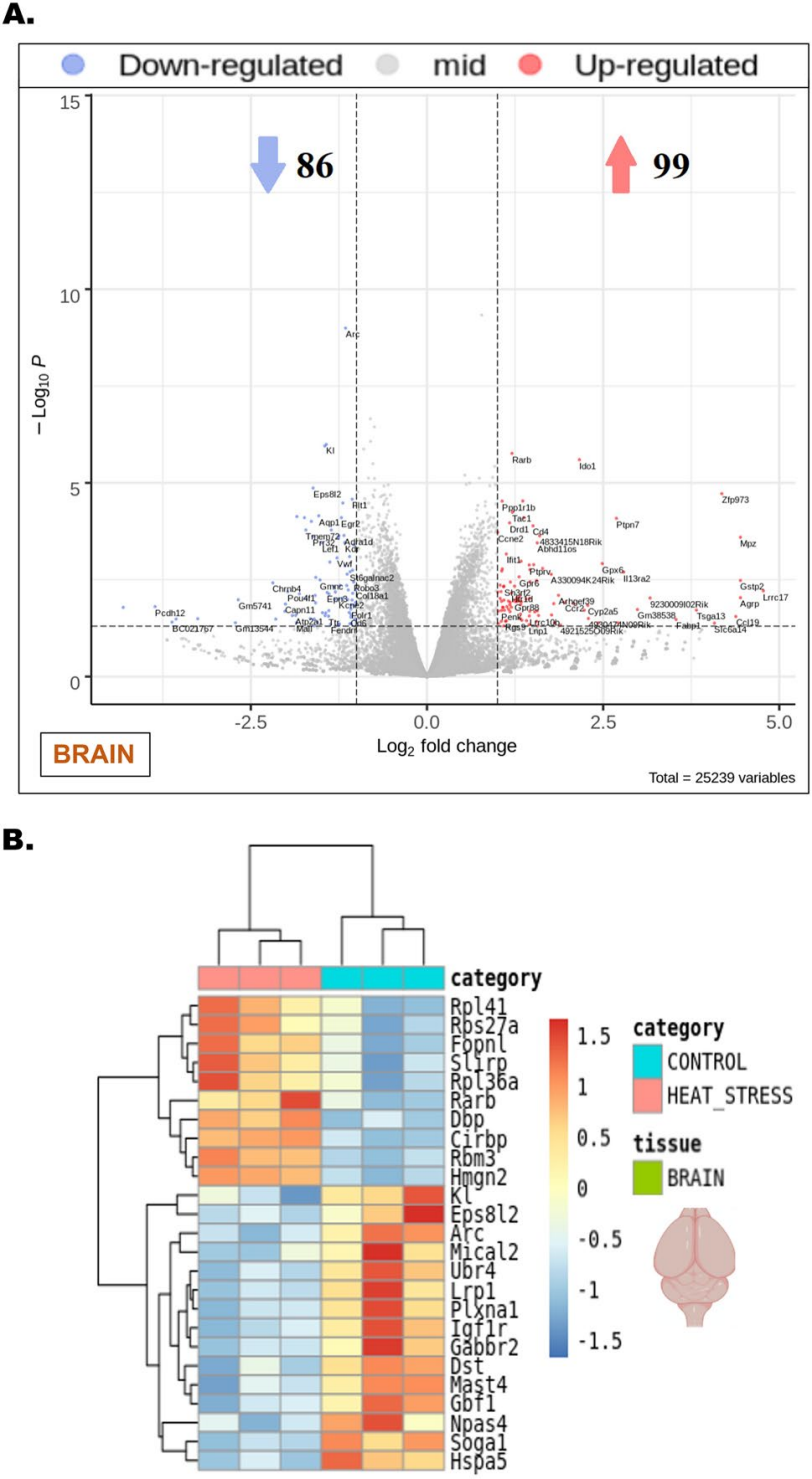
Heat stress-induced transcriptional alterations in the brain. (**A**) Volcano plot obtained from comparing the brain tissues of control and heat stress groups that were analyzed for differential gene expression changes through transcriptomic profiling, -Log_10_ P (*P*_*FDR*_) and *Log*_2_*fold changes* were plotted respectively along the ordinate and abscissa with a significance cut off set at *P*_*FDR*_ < 0.05, *Log*_2_ *fold change* > 1. The blue dots represented downregulated, and the red dots represented the upregulated genes. Genes represented in gray dots were laid in the zone of no significance. The total number of upregulated and downregulated genes was indicated with similar color arrowheads. (**B**) The heatmap generated from the brain tissue volcano plot was delineated with a *Log*_2_* fold change* gradient between 1.5 and − 1.5 where the extremities of red and blue signified up- and downregulations respectively. The same blue and red color code was set to represent the intensity of gene expression falling within the given gradient reflecting the *Log*_2_* fold change* of individual genes named parallel.

**Figure 3:**
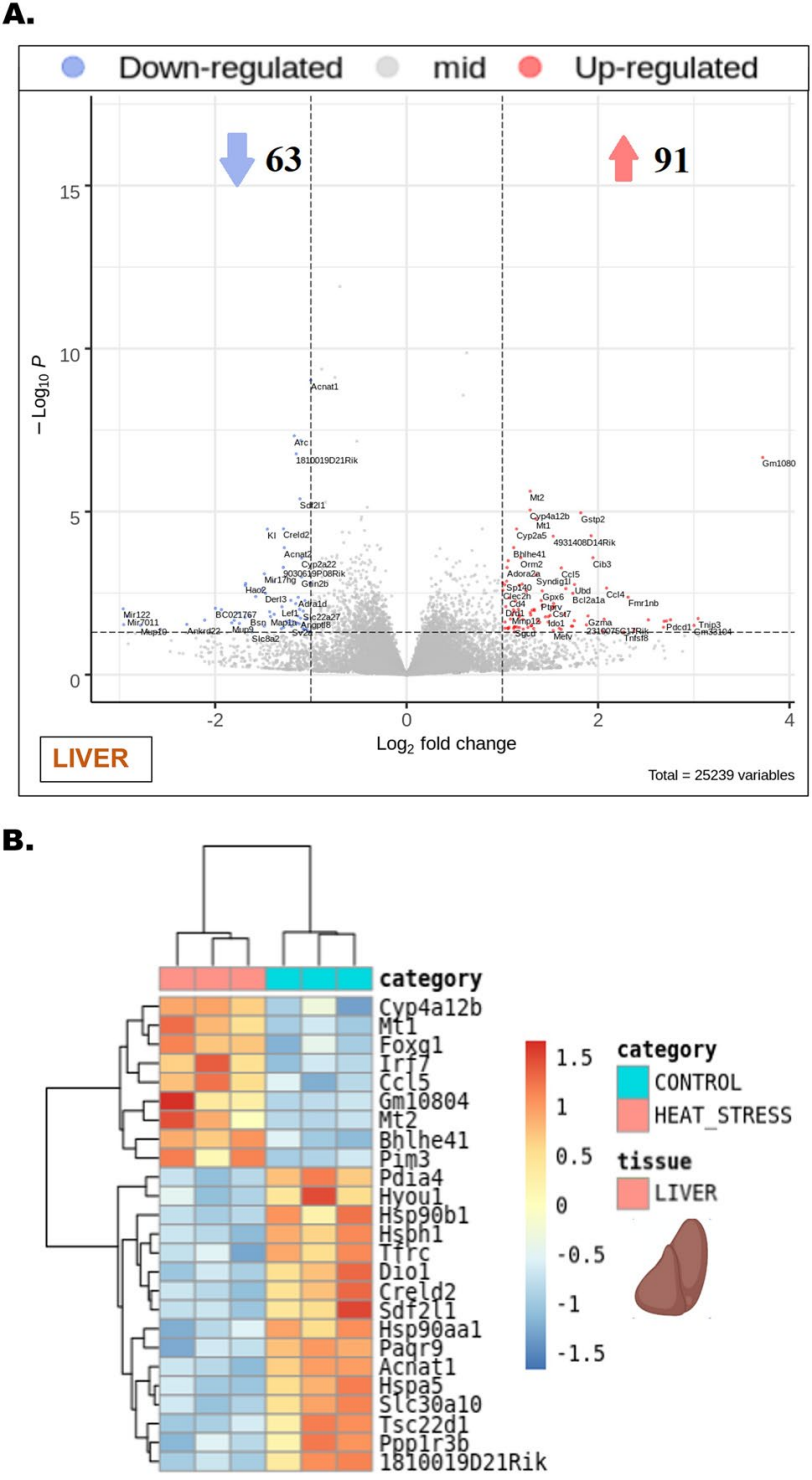
Heat stress-induced transcriptional alterations in the liver. (**A**) Volcano plot obtained from comparing the liver tissues of control and heat stress groups that were analyzed for differential gene expression changes through transcriptomic profiling, -Log_10_ P (*P*_*FDR*_) and *Log*_2_*fold changes* were plotted respectively along the ordinate and abscissa with a significance cut off set at *P*_*FDR*_ < 0.05, *Log*_2_ *fold change* > 1. The blue dots represented downregulated, and the red dots represented the upregulated genes. Genes represented in gray dots were laid in the zone of no significance. The total number of upregulated and downregulated genes was indicated with similar color arrowheads. (**B**) The heatmap generated from the liver tissue volcano plot was delineated with a *Log*_2_* fold change* gradient between 1.5 and − 1.5 where the extremities of red and blue signified up- and downregulations respectively. The same blue and red color code was set to represent the intensity of gene expression falling within the given gradient reflecting the *Log*_2_* fold change* of individual genes named parallel.

Upon exposure to heat stress, the cerebral tissues of the heat stress group manifested substantial alterations in the gene expression profile as illustrated in the volcano plot compared to the control (Fig. 2A). A total of 99 genes were significantly upregulated, while 86 were notably downregulated in response to the exposure of heat stress (Fig. 2A, *P*_*FDR*_ < 0.05, *Log*_2_ *fold change* > 1). The heat map, derived from transcriptomic profiling of brain lysates, portrayed the expression changes of genes such as *Rpl41, Fopnl, Rarb, Slirp, Hmgn2, Kl, Arc, Lrp1, Npas4, Mast4*, among others (Fig. 2B). Similarly, in hepatic tissues, heat stress resulted in a significant upregulation of 91 genes and downregulation of 63 genes, as indicated in the corresponding volcano plot (Fig. 3A, *P*_*FDR*_ < 0.05, *Log*_2_ *fold change* > 1). The pertinent heat map revealed alterations in the expression profile of genes including *Mt1, Mt2, Ccl5, Irf7, Pim3, Foxg1, Creld2, Acnat1, Pdia4*, and others within the liver tissues when subjected to heat stress (Fig. 3B).

### Heat stress-mediated gene expression changes were extrapolated in contributing pathways integral to organ-level pathological manifestations

**Figure 4:**
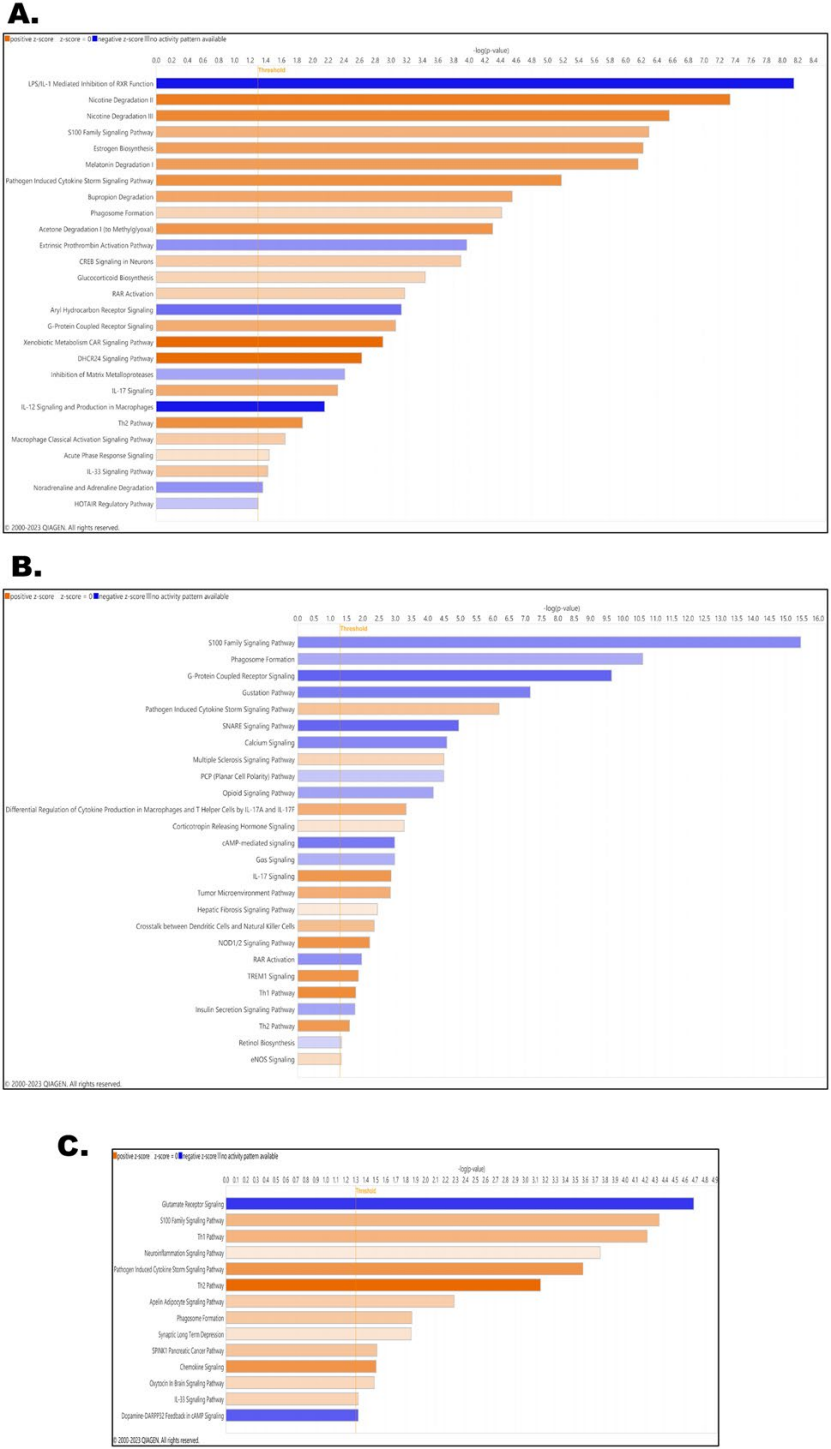
Heat stress-mediated gene expression changes were extrapolated in contributing pathways integral to organ-level pathological manifestations. (**A**) Biological pathways were predicted based on the differentially expressed gene list obtained by comparing the brain tissues from control and heat stress groups. Significance level set at *P*_*FDR*_ < 0.05, threshold at *-log(p value)* ~ *1.3*, *Log*_2_ *fold change* > 1. The gradients of blue and red colors represented the up- and downregulations of the pathways’ respectively. (**B**) Biological pathways were predicted based on the differentially expressed gene list obtained by comparing the liver tissues from control and heat stress groups. Significance level set at *P*_*FDR*_ < 0.05, threshold at *-log(p value)* ~ *1.3*, *Log*_2_ *fold change* > 1. The gradients of blue and red colors represented the up- and downregulations of the pathways’ respectively. (**C**) Biological pathways were predicted based on the commonly differentially expressed gene list obtained by comparing both the brain and liver tissues from control and heat stress groups. Significance level set at *P*_*FDR*_ < 0.05, threshold at *-log(p value)* ~ *1.3*, *Log*_2_ *fold change* > 1. The gradients of blue and red colors represented the up- and downregulations of the pathways’ respectively.

The pools of differentially expressed genes (DEGs), derived from comprehensive transcriptomic analyses, served as the foundation for predicting enriched biological pathways in the brain, liver, and shared DEGs indicative of the liver-brain axis under the influence of heat stress (Fig. 4). Within the brain, prominently upregulated pathways encompassed inflammatory S100B family signaling, IL-33 signaling associated with microglial activation, degradation pathways for dopamine, nicotine, acetone, bupropion, and melatonin, Th2 signaling, xenobiotic metabolism CAR signaling, IL-17 signaling, DHCR24 signaling, G-protein coupled receptor signaling, pathogen-induced cytokine storm signaling, and acute phase response signaling, among others. Conversely, downregulated pathways included aryl hydrocarbon receptor signaling (AhR), IL-12 signaling, IL-1 mediated inhibition of RXR signaling, inhibition of matrix metalloprotease signaling, extrinsic prothrombin activation signaling, and several other pathways significant for inflammatory pathology (Fig. 4A; *P*_*FDR*_ < 0.05, the threshold at *-log(p value)* ~ *1.3*, *Log*_2_ *fold change* > 1). In the liver, tissues exposed to heat stress, the upregulated pathways comprised of IL-17 signaling, NOD1/2 signaling associated with liver injury, TREM1 signaling of hepatic inflammation, tumor microenvironment signaling, Th1 signaling, and eNOS signaling. Meanwhile, downregulated pathways included G-protein coupled receptor signaling, cyclic AMP (cAMP) mediated signaling, retinoic acid receptor signaling (RAR), SNARE signaling, calcium, and opioid signaling, among others (Fig. 4B; *P*_*FDR*_ < 0.05, the threshold at *-log(p value)* ~ *1.3*, *Log*_2_ *fold change* > 1). The predicted pathways of the liver-brain axis exhibited upregulation in chemokine signaling, Th1 and Th2 signaling, neuroinflammation signaling, synaptic long-term depression signaling, cytokine storm signaling, apelin adipokine signaling, and more. Conversely, downregulated pathways included glutamate signaling and dopamine-DARPP32 feedback in cAMP signaling (Fig. 4C; *P*_*FDR*_ < 0.05, the threshold at *-log(p value)* ~ *1.3*, *Log*_2_*fold change* > 1).

### Predicted pathway circuits unveiled a neurological disease network within the brain and an inflammatory control network within the liver tissues following exposure to heat stress

**Figure 5:**
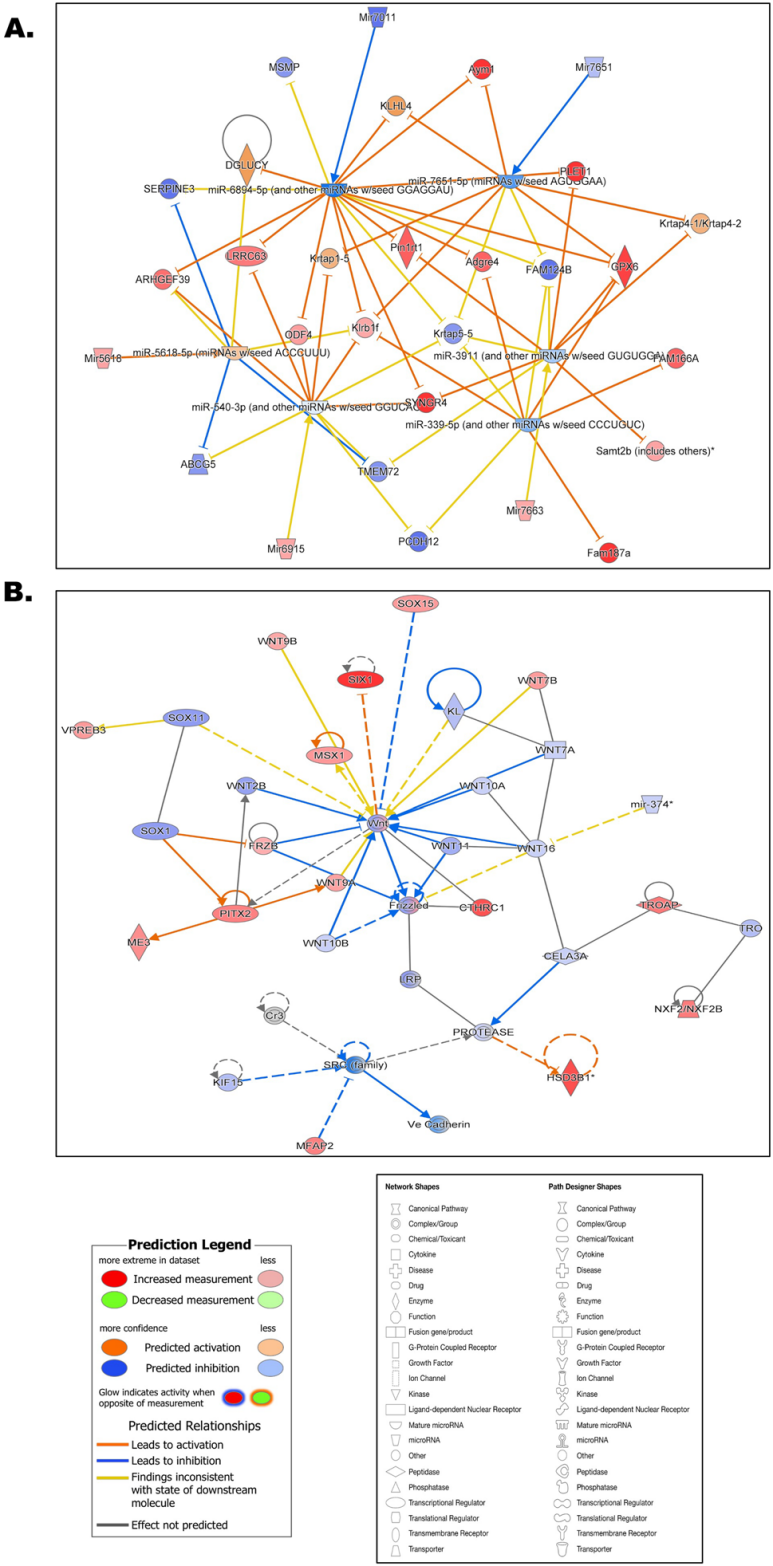
Predicted pathway circuits unveiled a neurological disease network within the brain and an inflammatory control network within the liver tissues following exposure to heat stress. (**A**) Pathway circuit predicted based on the projected pathways obtained by comparing the brain tissues from control and heat stress groups. Each shape represented different biological kinds of molecules and pathway components and each line connector style represented activation/inhibition status. Detailed legends of prediction, network, and path designer shapes were included in the figure. (**B**) Pathway circuit predicted based on the projected pathways obtained by comparing the liver tissues from control and heat stress groups. Each shape represented different biological kinds of molecules and pathway components and each line connector style represented activation/inhibition status. Detailed legends of prediction, network, and path designer shapes were included in the figure.

Anticipated enhancements in the measurement and activation of gene products, such as Synaptogyrin 4 (*Syngr4*), Kelch-like protein 4 (*Klhl4*), *Gpx6, Plet1, Adgre4, Pin1rt1, Fam187a, Lrrc63, Am166a, Klrb1f., Mir5618, Mir6915, Mir7663, Odf4*, were followed by diminished measurements and inhibition of gene products, namely Protocadherin 12 (*Pcdh12*), Transmembrane protein 12 (*Tmem12*), *Serpine3, Tmem72, Fam124b, Krtap5-5, Msmp, Mir7011, Mir7651, Abcg5*, observed in the neurological disease network within the brain tissues exposed to heat stress (Fig. 5A). The inflammatory control network within the liver tissues revealed a distinct set of patterns. Increased measurement and activation of gene products included *Six1, Pitx2, Cthrc1, Me3, Mfap2, Msx1, Wnt7b, Wnt9b, Hsd3b1* etc. whereas decreased measurement and inhibition was observed in *Sox1, Sox11, Lrp, Frizzled, Wnt10a, Wnt10b, Kl, Kif15, Cadherin, Mir374* etc. (Fig. 5B).

### Altered functional interactome emerged in both brain and liver tissues upon exposure to heat stress

**Figure 6:**
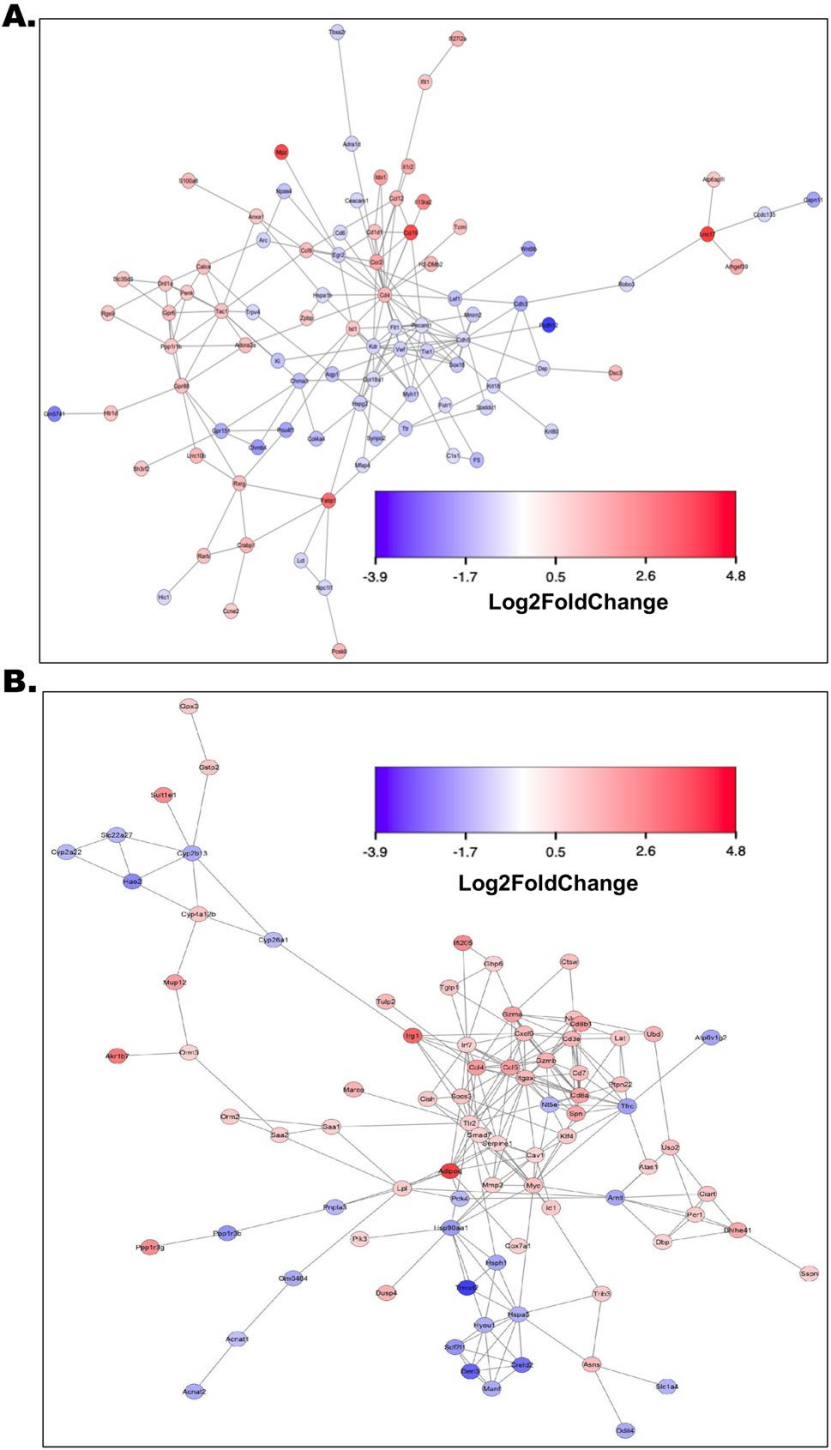
Altered functional interactome emerged in both brain and liver tissues upon exposure to heat stress. (**A**) Interactome was predicted based on the differentially expressed genes obtained from transcriptomic profiling and then by comparing the brain tissues from control and heat stress groups. Interaction intensities were signified by possible upregulations and downregulations of the respective gene clusters with a *Log*_2_* fold change* gradient between − 3.9 and − 4.8 where the extremities of red and blue indicated up- and downregulations respectively. The same blue and red color code was set to mark the genes in clusters falling within the given gradient, gene names were provided in each representing shape. The connector line indicated the possible biological strength and interconnections of the interacting gene products. (**B**) Interactome was predicted based on the differentially expressed genes obtained from transcriptomic profiling and then by comparing the liver tissues from control and heat stress groups. Interaction intensities were signified by possible upregulations and downregulations of the respective gene clusters with a *Log*_2_* fold change* gradient between − 3.9 and − 4.8 where the extremities of red and blue indicated up- and downregulations respectively. The same blue and red color code was set to mark the genes in clusters falling within the given gradient, gene names were provided in each representing shape. The connector line indicated the possible biological strength and interconnections of the interacting gene products.

In cerebral tissues, distinct alterations were observed within several gene clusters of the predicted interactome. Notably, there were prominent upregulations in immune clusters, encompassing CD19, IL13RA2, and CD4, followed by the S100B inflammation cluster, LRRC17, NF-κB activation, and MPZ-mediated demyelination response. Conversely, downregulations were observed in clusters associated with PCDH12-mediated neuronal differentiation, melatonin receptors, and GM5741 (Fig. 6A). In liver tissues, the upregulations were projected in the immune cluster involving CD8A, CCL4, CCL5, CD7, LRG1, IRF7, CXCL9, KLF4, along with Adiponectin-mediated liver injury response, ER stress response, Aldo–keto reductases, and AKR1B7. Simultaneously, several downregulation of genes were predicted in clusters related to metabolic rhythm, HAO2-mediated oxidative stress response and ARNTL-mediated steatosis (Fig. 6B).

### The liver-brain connection in heat stress pathologies was projected through a shared predictive pathway circuit and interactome

**Figure 7:**
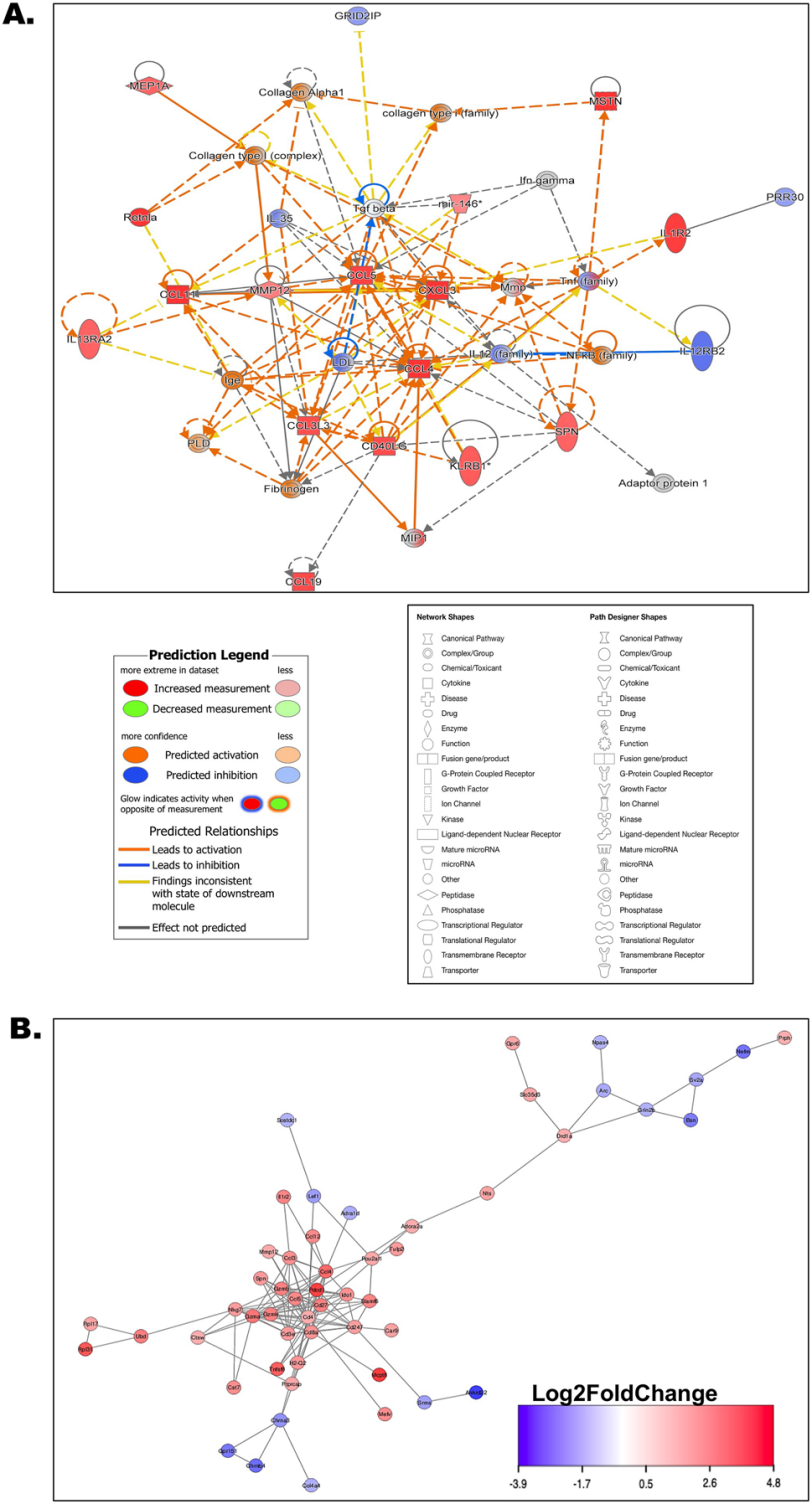
The liver-brain connection in heat stress pathologies was projected through a shared predictive pathway circuit and interactome. (**A**) Pathway circuit predicted based on the projected pathways obtained by comparing both brain and liver tissues from control and heat stress groups. Each shape represented different biological kinds of molecules and pathway components and each line connector style represented activation/inhibition status. Detailed legends of prediction, network, and path designer shapes were included in the figure. (**B**) Interactome was predicted based on the differentially expressed genes obtained from transcriptomic profiling and then by comparing both brain and liver tissues from control and heat stress groups. Interaction intensities were signified by possible upregulations and downregulations of the respective gene clusters with a *Log*_2_* fold change* gradient between − 3.9 and − 4.8 where the extremities of red and blue indicated up- and downregulations respectively. The same blue and red color code was set to mark the genes in clusters falling within the given gradient, gene names were provided in each representing shape. The connector line indicated the possible biological strength and interconnections of the interacting gene products.

A comparative analysis of liver and brain transcriptomes identified commonly differentially expressed genes, thereby elucidating the liver-brain connection. The predictive outcomes indicated potential enhancements in the measurement and activation of genes such as *Ccl3, Ccl4, Ccl5, Ccl11, Ccl19, Cxcl3, IL1r2, IL13ra2, Mep1a, Klrb1, Mip1, Retnla, Mep1a, Spn, Nf-κb family, Mmp12, Mir146, Mstn, Ige, Fibrinogen, Pld and Collagen α1.* Conversely, decreased measurements and inhibition of gene products were observed for *Prr30, IL35, IL12 family, and IL12rb2* (Fig. 7A). In the predicted interactome, significant upregulations were observed within gene clusters of TNFSF8, PDCD1, CCL4, RPL31, MCPT8, IL1R2, CCL12, GPR6, ADORA2A, CD8A, and GZMA, while downregulations were predicted for clusters of NEFM, BSN, NPAS4, ANKRD22, GPR151, CHRNB4, ADRA1D and LEF1 (Fig. 7B).

### Expression fold changes of the top genes observed in the brain and liver unveiled potential validatory mediators of the liver-brain axis upon exposure to heat stress

**Figure 8:**
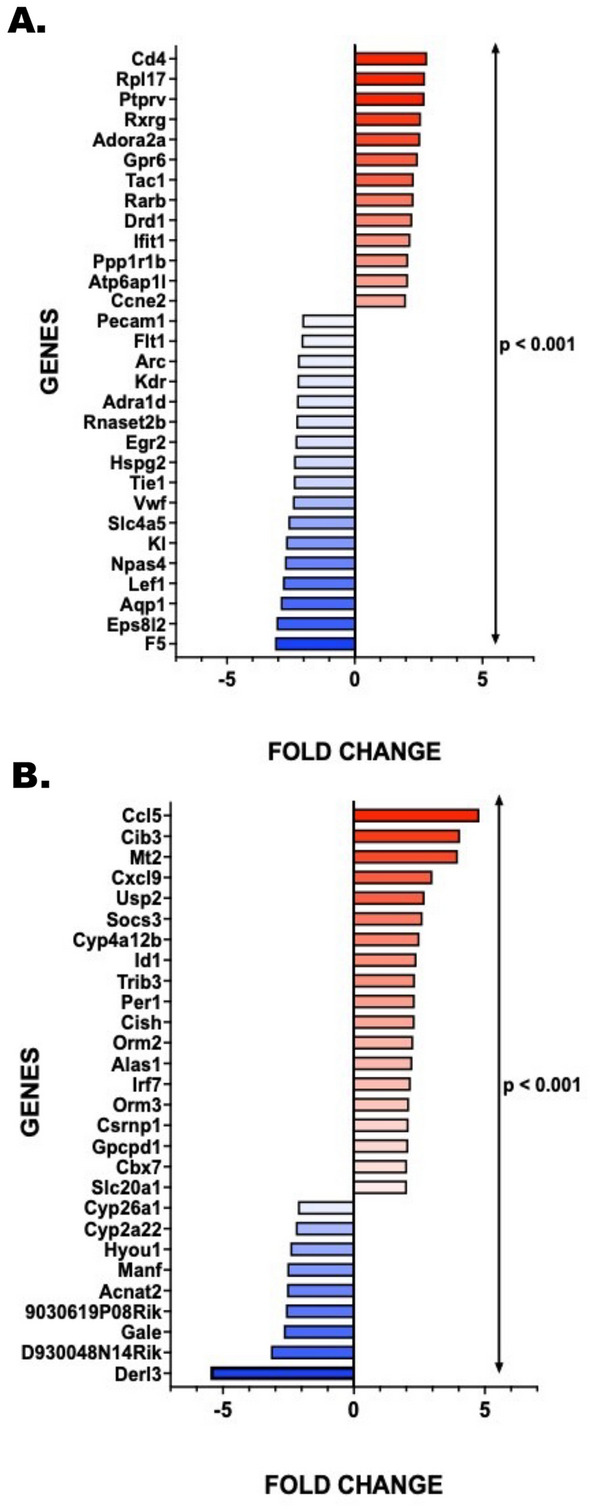
Expression fold changes of the top genes observed in the brain and liver unveiled potential validatory mediators of the liver-brain axis upon exposure to heat stress. (**A**) Heat Stress-induced relative mRNA expression changes (18S rRNA normalized) of the top genes in brain samples were converted from log2 fold changes based on the transcriptomic data plotted in bar graphs representing each gene (Control representing the baseline, 0). Negative fold changes were indicated in the blue color gradient whereas positive fold changes were indicated in the red gradient. All *p* values were derived through an unpaired t-test, with the significance level set at *p* < 0.05. (**B**) Heat Stress-induced relative mRNA expression changes (18S rRNA normalized) of the top genes in liver samples were converted from log2 fold changes based on the transcriptomic data plotted in bar graphs representing each gene (Control representing the baseline, 0). Negative fold changes were indicated in a blue color gradient whereas positive fold changes were indicated in a red color gradient. All *p* values were derived through an unpaired t-test, with the significance level set at *p* < 0.05.

The transcriptomics data was further screened to identify key genes that could mediate the effects of heat stress. This was done by examining the gene list showing differential expression due to heat stress. The changes in gene expression were quantified using log_2_ fold changes, which were then converted to expression fold changes. Several genes were found to be upregulated in the brain, including *Cd4, Rpl, Ptprv, Adora2a, Gpr6, and Tac1,* among others. Conversely, genes such as *Eps8l2, F5, Aqp1, Npas4, Slc4a5, Pecam1,* and others were downregulated (Fig. 8A; *p* < 0.001). When comparing the liver data from the control and heat stress groups, a significant set of genes were identified that could potentially act as mediators. Some of these mediators could potentially reach the brain via the systemic route and exert effects on the neuronal circuits. The upregulated genes included acute phase reactants like *Orm2*, chemokines such *as Ccl5, Cxcl9,* and other crucial non-mediators like *Cib3, Usp2, Socs3, Irf7,* etc. On the other hand, genes like *Derl3, Gale, Acnat2, Manf,* and others were found to be downregulated (Fig. 8B; p < 0.001).

### Heat stress augmented the secretion of hepatic ORM2 and its abundance in the hippocampus along with the frontal cortex, likely traversing through leaky Blood–Brain-Barrier (BBB)

**Figure 9:**
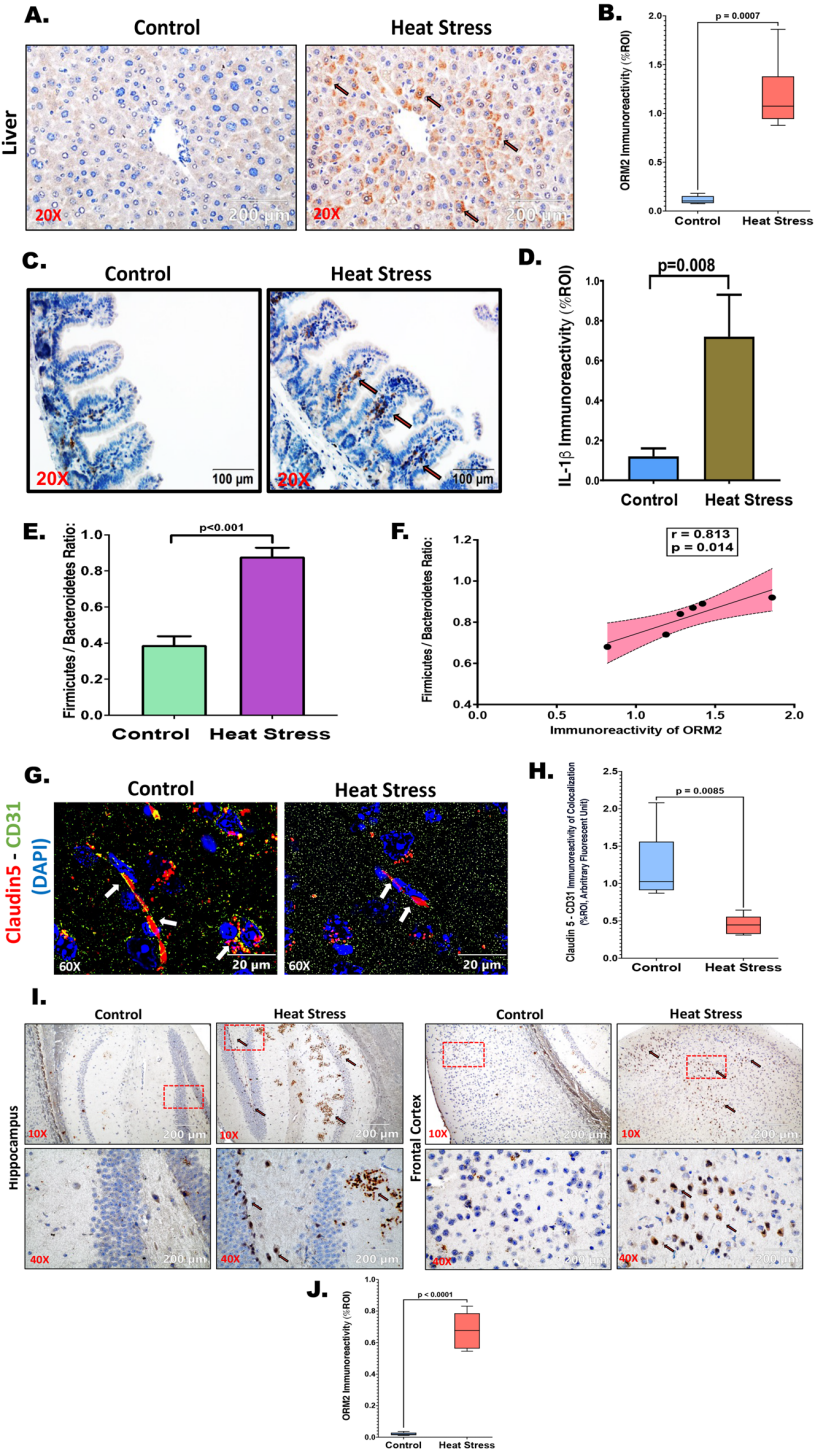
Heat stress augmented the secretion of hepatic ORM2 and its abundance in the hippocampus along with the frontal cortex, traversing through leaky Blood–Brain-Barrier (BBB). (**A**) ORM2 immunoreactivity was shown by immunohistochemistry in liver sections from Control and Heat Stress mouse groups; Images were taken at 20X magnification and displayed with a scale of 200 μm. (**B**) ORM2 immunoreactivity in both groups was measured as arbitrary light units from six separate microscopic fields and plotted along the ordinate. All *p* values were derived through an unpaired t-test, with the significance level set at *p* < 0.05. (**C**) IL-1β immunoreactivity was shown by immunohistochemistry in small intestinal sections from Control and Heat Stress mouse groups; Images were taken at 20X magnification and displayed with a scale of 100 μm. (**D**) IL-1β immunoreactivity in both groups was measured as arbitrary light units from six separate microscopic fields and plotted along the ordinate. All *p* values were derived through an unpaired t-test, with the significance level set at *p* < 0.05. (**E**) The bar graph was plotted as the difference in ratio value of the relative abundance of the gut microbial phyla Firmicutes and Bacteroidetes compared between control and heat stress groups. The *p* value was derived through an unpaired t-test, with the significance level set at *p* < 0.05. (**F**) A correlation analysis was conducted between the gut dysbiosis marker Firmicutes/Bacteroidetes ratio and the abundance of ORM2 in liver tissues. The correlation plot includes Pearson's linear regression depicted in red, along with a 95% confidence interval. (**G**) Claudin5 (red) and CD31 (green) were dual labeled through immunofluorescence staining with colocalization (yellow) observed at 60X (oil) magnification. The images were presented with a scale of 20 μm in the brain sections from both the Control and Heat Stress mouse groups. (**H**) Claudin5-CD31 colocalization (yellow) events in both groups were quantified as arbitrary fluorescent units from six distinct microscopic fields and plotted along the ordinate. All *p* values were derived through an unpaired t-test, with the significance level set at *p* < 0.05. (**I**) ORM2 immunoreactivity was shown by immunohistochemistry in brain sections (hippocampus and frontal cortex) from Control and Heat Stress mouse groups; Images were taken at 10X, 40X magnification displayed with the scale of 200 μm. (**J**) ORM2 immunoreactivity in both groups was measured as arbitrary light units from six separate microscopic fields and plotted along the ordinate. All *p* values were derived through an unpaired t-test, with the significance level set at *p* < 0.05.

Immunohistochemical analysis of liver slices showed a marked augmentation in Orosomucoid 2 (ORM2) immunoreactivity, signifying an escalated release of this protein at the tissue level within the liver of the heat stressed group (Fig. 9A,B; *p* < 0.001). Previous research has established a linkage between ORM2 release from the liver and its involvement in intestinal inflammation and gut dysbiosis^30^. Consequently, IL-1β immunoreactivity was assessed as an indicator of intestinal inflammation within the experimental mouse cohorts. IL-1β immunoreactivity exhibited a substantial increase in the intestinal sections from the heat stress group compared to the control (Fig. 9C,D; *p* = 0.008). Notably, heat stress-induced gut dysbiosis has been acknowledged as a contributing factor to intestinal inflammation^31,32^. Therefore, an investigation was performed into the correlation between gut dysbiosis and intestinal inflammation concerning the release of ORM2 in the liver. The Firmicutes to Bacteroidetes ratio has long been recognized as a marker of gut dysbiosis in the context of hepatic inflammation and diseases^33–35^. Hence, the whole metagenome sequencing of fecal samples from the control and heat stress mouse groups were performed. The data elucidated a significant increase in the Firmicutes to Bacteroidetes ratio in the heat stress group compared to the control (Fig. 9E; *p* < 0.001). Given the previously established correlation between ORM2 abundance and gut dysbiosis, a correlation plot was constructed, revealing a significant positive correlation between the Firmicutes to Bacteroidetes ratio and ORM2 immunoreactivity in liver tissues of the heat stress mouse group (Fig. 9E; *p* = 0.014, *r* = 0.813). Subsequently, the integrity of the blood–brain barrier (BBB) was examined in brain sections of the respective mouse groups. Immunofluorescence analysis showed BBB disruption, quantified in terms of Claudin 5 (Red) and CD31 (Green) colocalization events (Yellow) counterstained with DAPI for nuclear imaging (Fig. 9G; *p* < 0.001). Colocalization was markedly diminished in the heat stress group compared to the control, suggesting a potential disruption of the BBB (Fig. 9H; *p* = 0.0085). ORM2 protein secretion primarily emanated from hepatocytes^36,37^. Past investigations have delineated the role of ORM2 in neuroinflammation and ischemic stroke events^38,39^. Consequently, the abundance of ORM2 was evaluated in brain sections, revealing a significant increase in ORM2 immunoreactivity in the hippocampus and frontal cortex regions of the brain in the heat stress group (Fig. 9A,B; *p* < 0.001). Intriguingly, the brain transcriptome analysis disclosed no ORM2 mRNA fold changes in the heat stress group when compared to the control. Despite ORM2 being predominantly secreted by the liver, its heightened abundance in the brain suggests increased secretion from hepatocytes during heat stress, possibly reaching brain tissues through a leaky blood–brain barrier via the systemic route.

### Heat stress-induced transcriptomic alterations together with acute phase response ensued neural, hepatic, and systemic inflammation further debilitating synaptic plasticity

**Figure 10:**
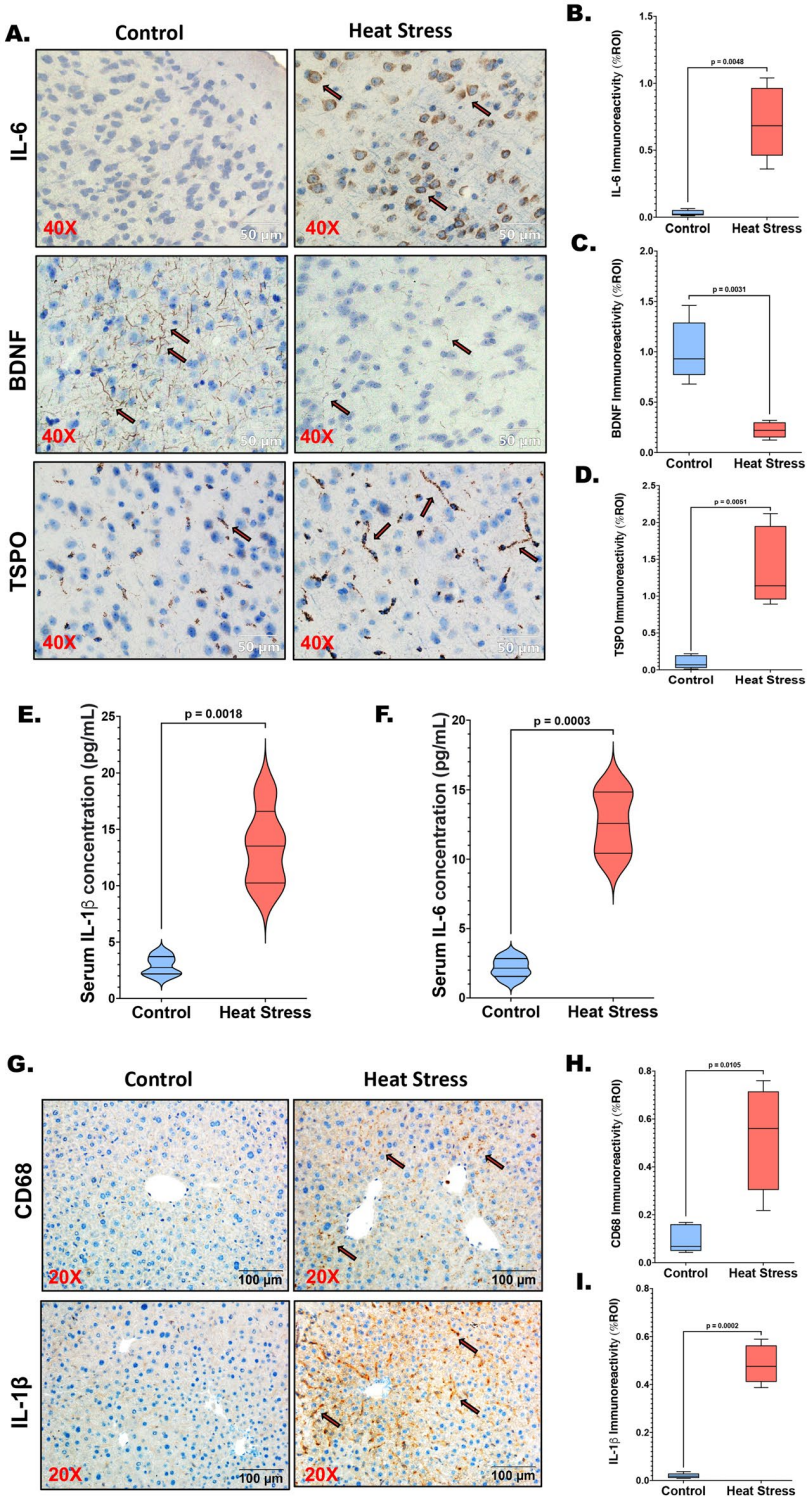
Heat stress-induced transcriptomic alterations together with acute phase response ensued in neural, hepatic, and systemic inflammation debilitating synaptic plasticity. (**A**) IL-6, BDNF, and TSPO immunoreactivities were shown by immunohistochemistry in the brain (frontal cortex) sections from Control and Heat Stress mouse groups; Images were taken at 40X magnification displayed with the scale of 50 μm. (**B**) IL-6 immunoreactivity in both groups was measured as arbitrary light units from six separate microscopic fields and plotted along the ordinate. All *p* values were derived through an unpaired t-test, with the significance level set at *p* < 0.05. (**C**) BDNF immunoreactivity in both groups was measured as arbitrary light units from six separate microscopic fields and plotted along the ordinate. All *p* values were derived through an unpaired t-test, with the significance level set at *p* < 0.05. (**D**) TSPO immunoreactivity in both groups was measured as arbitrary light units from six separate microscopic fields and plotted along the ordinate. All *p* values were derived through an unpaired t-test, with the significance level set at *p* < 0.05. (**E**) Serum IL-1β level in pg/mL was graphed in violin plots from Control and Heat Stress mouse serum samples (n = 3). All *p* values were derived through an unpaired t-test, with the significance level set at *p* < 0.05. (**F**) Serum IL-6 level in pg/mL was graphed in violin plots from Control and Heat Stress mouse serum samples (n = 3). All *p* values were derived through an unpaired t-test, with the significance level set at *p* < 0.05. (**G**) CD68 and IL-1β immunoreactivities were shown by immunohistochemistry in liver sections from Control and Heat Stress mouse groups; Images were taken at 20X magnification and displayed with a scale of 100 μm. (**H**) CD68 immunoreactivity in both groups was measured as arbitrary light units from six separate microscopic fields and plotted along the ordinate. All *p* values were derived through an unpaired t-test, with the significance level set at *p* < 0.05. (**I**) IL-1β immunoreactivity in both groups was measured as arbitrary light units from six separate microscopic fields and plotted along the ordinate. All *p* values were derived through an unpaired t-test, with the significance level set at *p* < 0.05.

Immunohistochemical analysis conducted on brain sections aimed to explore the pleiotropic cytokine IL-6, the synaptic plasticity marker Brain-Derived Neurotrophic Factor (BDNF), and the microglial activation marker Translocator Protein (TSPO) (Fig. 10A). Within the heat stress group, a noteworthy augmentation in IL-6 immunoreactivity was discerned (Fig. 10A,B; *p* = 0.0048), while the immunoreactivity of BDNF exhibited a significant decrease (Fig. 10A,C; *p* = 0.0031). Additionally, as assessed by TSPO immunoreactivity, microglial activation was significantly heightened in the heat stress group compared to the control (Fig. 10A,D; *p* = 0.0051). The systemic level of the proinflammatory cytokine IL-1β was quantified via ELISA using serum samples from the control and heat stress mice groups, revealing a substantial elevation in the systemic IL-1β level within the heat stress group (Fig. 10E; *p* = 0.001). Furthermore, a significant increase in the systemic level of IL-6 was also evident in the heat stress group in comparison to controls (Fig. 10F; *p* < 0.001). Immunohistochemistry conducted on liver slices from the control and heat stress mouse groups showed a marked escalation in the immunoreactivities of the Kupffer cell activation marker CD68 (Fig. 10G,H; *p* = 0.0105) and the proinflammatory cytokine IL-1β (Fig. 10G,I; *p* < 0.001) in the heat stress group.

## Discussion

This study is the first report of a molecular investigation using genomic approaches of heat stress pathophysiology, focusing on the concerted actions of brain and liver. Further, the present study focused on the molecular pathways of the gut-liver-brain axis, linking intestinal pathology and gut dysbiosis. Employing multiorgan transcriptomic profiling, it identified common heat stress-affected genes in the brain and liver. Rigorous bioinformatics analysis unveiled significant transcriptome changes and identified enriched pathways. The study uniquely identified liver-derived mediators connecting the gut-brain-liver axis and intricately correlated with heat stress pathophysiology, marking an effort to unravel organ interplay during heat stress, advancing molecular insights into the pathophysiology of adverse heat events.

Top differentially expressed genes, like *Rpl41*, upregulated in the brain under heat stress are linked to ATF4 phosphorylation, oxidative stress, and neuronal degeneration, notably in Alzheimer’s and Parkinson's diseases^40^. Another gene, *Rarb* was also found to be upregulated in heat-stress brain tissues, and gain-of-function mutations of *Rarb* are associated with intellectual disability and progressive motor impairment, indicating a potential role of *Rarb* in brain function and development^41^. *Hmgn2* is upregulated in cerebral tissue upon heat stress and plays a specific role in the brain, maintaining chromatin accessibility during corticogenesis, crucial for adult neurogenesis and synaptic plasticity^42^. Heat stress in our study was observed to downregulate *Npas4*, a brain-expressed transcription factor crucial for memory and inhibitory synapse formation, with deficiency linked to cognitive deficits and implications in neuroprotection against stress, offering potential therapeutic insights for conditions like epilepsy, Alzheimer's^43,44^. Furthermore, Npas4 significantly downregulated in brain tissues from the heat stress group was reported previously to serve in the maintenance of the blood–brain barrier integrity, and its deficiency compromises neuroprotection^45–47^ which is supportive of this study’s observations of the leaky blood–brain barrier and deteriorating synaptic plasticity in the heat stress exposed mice. The enriched pathways observed in the brain under heat stress strongly highlighted the activation of S100B signaling, a central nervous system calcium-binding protein concentrated in astrocytes, that serves as a reliable biomarker of neural distress and is implicated in neuropathology. In brain inflammation, the above molecule acts as both a pro-inflammatory cytokine and a Damage-Associated Molecular Pattern (DAMP), displaying concentration-dependent effects on neurons, ranging from neurotrophic support to inducing apoptosis^48,49^. Another pathway predicted in brain tissues of the heat stress group was the activation of Interleukin-17 (IL-17) signaling, which is associated with disrupting the blood–brain barrier, fostering neuroinflammation, and contributing to tissue damage in neuroinflammatory conditions of ischemic brain injury. Its impact on various neural cell types, including microglia, endothelial cells, astrocytes, and neurons, triggers inflammatory responses and poses a risk of tissue damage^50^. Interleukin-33 (IL-33) was also observed to have enriched in brain lysates of the heat stress group known to exhibit roles in brain inflammation. This may act as a pro-inflammatory mediator that can activate cells such as astrocytes and microglia, potentially leading to sustained inflammation, neuronal damage, and cognitive impairment in neuroinflammatory pathology^51,52^. Additionally, downregulation of the Interleukin-12 (IL-12) signaling has been observed in heat stress-induced neuronal pathways; IL-12 is associated with inducing neuroprotection and shaping the trophic factor milieu within the inflamed central nervous system, with neurons being the dominant source of trophic factors^53^. Neurological disease network extrapolations revealed genes like Synaptogyrin 4 which is novel in depicting neuronal distress while other proteins of the same family were previously correlated with synaptic plasticity factors under various neurological disease pathology^54,55^. Serpine 3, was inhibited in heat-stressed brain tissues and is known to hasten the neuronal apoptosis and more severe impairments of spatial learning and memory following a hippocampal injury^56^. Observations from the transcriptomic profiling and downstream bioinformatic analyses of brain tissues comparing control and the heat stress groups are in concordance with the functional validations indicating neuroinflammation (IL-6 release, microglial activation, TSPO) and cognitive decline (decrease in BDNF) marked in the heat stress group.

Upregulated genes in the liver due to heat stress comprised of *Ccl5*, or *Rantes* (Regulated upon Activation, Normal T Cell Expressed and Secreted), a well-known hepatic chemokine influencing immune cell recruitment in liver disease. It activates T cell proliferation and cytokine production, contributing to inflammatory responses, hepatic fibrosis, and early stages of metabolic dysfunction-associated steatotic liver disease (MASLD) by inducing steatosis through the Ccr5 receptor^57,58^. *Creld2* or Cysteine-rich with EGF-like domains 2 was also upregulated in the liver lysates of the heat stress group and is reported to be crucial for liver metabolism homeostasis, particularly in the unfolded protein response (UPR). It also acts as an ER-stress inducible gene, enhancing protein folding, and its deficiency is associated with dysregulated UPR, and hepatic steatosis, and impacts energy expenditure, emphasizing its key role in liver health and function^59^. *Irf7* (Interferon Regulatory Factor 7) was upregulated, which is also crucial for IL-1β release and concomitant inflammatory response in the liver^60^. Pathway enrichment study identified the activation of NOD1/NOD2 in liver tissues of the heat stress group which triggers proinflammatory and type I interferon responses, contributing to liver injury. These receptors play a role in recognizing bacterial pathogens, inducing liver inflammation, and have associations with innate immune activation in metabolic diseases, with studies suggesting that blocking NOD1 can be protective against tissue injury^61,62^. TREM-1 (Triggering Receptor Expressed on Myeloid Cells 1) signaling was also activated in heat-stressed liver tissues and is implicated in liver inflammation and fibrosis by promoting hepatic inflammation, activating stellate cells, and serving as a master regulator of Kupffer cell activation and linked to inflammation, lipid accumulation, fibrosis, and tumor progression in liver-related diseases^63^. Interleukin-17 (IL-17) signaling activation was notable in the liver transcriptome of the heat stress group, which is linked to liver inflammation and fibrosis, with elevated levels observed in liver diseases, inducing immune cell infiltration, damage, inflammation, and fibrosis. Targeting the IL-17 axis is considered a potential therapeutic approach for treating liver-related conditions^64,65^. Interactome mapping in the liver lysates from the heat stress group showed an array of significant interactions compared to the controls. Projected upregulations were observed in the adipokine LRG1 (Leucine-rich alpha-2-glycoprotein 1) associated with liver diseases, notably in obesity-induced hepatosteatosis and insulin resistance. Its endocrine action, including suppressing fatty acid β-oxidation and promoting de novo lipogenesis, contributes to hepatosteatosis. At the same time, LRG1 also influences Kupfer cell activation in the liver and enhances TGF-β1 signaling, suggesting a significant role in liver metabolism and diseases associated with obesity^66,67^. The Krüppel-like factor 4 (KLF4) transcription factor has been associated with various physiological and pathological processes in the liver and was also found to be upregulated in the liver interactome of the heat stress group is known to augment TGF-β1 expression and liver inflammation, indicating a potential role in modulating the fibrotic response in organ systems including liver fibrosis^68^. The observations from the transcriptomic profiling and downstream bioinformatic analyses of the liver tissues comparing control and the heat stress groups are congruent with the functional studies conducted. Validations revealed a correlative hepatic inflammation (Kupfer cell activation, CD68 immunoreactivity) and IL-1β release, suggesting the onset of hepatic inflammation.

The thorough bioinformatic analyses further revealed the liver-brain connection in heat stress based on the commonly differentiated genes in both organs. The liver-brain axis is a vital communication network influencing human health, connecting the gut, liver, and brain. It plays a role in various disease conditions involving endocrine, humoral, metabolic, and immune pathways^69^. The impact of climate change on neurological diseases and gastrointestinal health, including the liver-brain axis, has been highlighted, with potential implications for metabolic liver diseases and associated neuropathologies^70^. The increased activity in chemokine signaling, Th1 and Th2 signaling, neuroinflammation signaling, synaptic long-term depression signaling, adipokine signaling, etc. were observed in the enriched pathways of the Liver-Brain axis upon exposure to heat stress. An array of cytokines such as CCL3, CCL4, CCL5, IL-35, IL-12, etc., different acute phase responders like ORM2, ORM3, etc., and other intricate non-mediators were identified. Among these, the ORM2, a liver-produced hepatokine, plays a crucial role in maintaining hepatic and systemic lipid balance and is implicated in regulating de novo lipogenesis^71^. ORM2 levels are associated with MASLD, while studies suggest its potential as a therapeutic target for liver diseases, particularly those related to lipid metabolism and steatohepatitis^36^. Additionally, ORM2 has shown promise as a biomarker for liver diseases, contributing to diagnostics and monitoring, emphasizing its significance in liver health and potential for therapeutic interventions^72^. Following the literature, our observations also revealed a significant increase in the production of hepatic ORM2 in the liver tissues of the heat stress group, compared with the control group, indicating heat stress elevated ORM2 production in the liver. Existing literature described the link between ORM2 release from the liver and its involvement with intestinal inflammation and gut dysbiosis^30^. Our results revealed the intestinal inflammation caused due to exposure to heat stress and concomitant events of gut dysbiosis measured through the alteration of Firmicutes to Bacteroidetes ratio. The Firmicutes to Bacteroidetes ratio is a gold-standard marker of gut dysbiosis^33,34^. Heat stress is well characterized in inducing gut dysbiosis and has been proven robust in causing intestinal inflammation^31^. Our results indicated a positive correlation plotted with gut dysbiosis and hepatic ORM2 production. Therefore, our data successfully delineated the compelling correlation between intestinal inflammation, gut dysbiosis, and hepatic ORM2 release in the context of heat stress-induced pathophysiology. This tended to justify the cause of augmented ORM2 production from the liver under heat stress. Also, systemic markers of inflammation, such as the levels of IL-6 evident in our data sets, pointed to the acute phase response in heat stress, which is consistent with existing literature^73,74^. Past studies have described the role of ORM2 in neuroinflammation and ischemic stroke events, and the existence of astrocyte-produced ORM2 was also deciphered^38,39^. Our results revealed a significant rise in ORM2 abundance in the brain's hippocampus and frontal cortex regions upon exposure to heat stress. However, the transcriptomic profiling of the brain tissues showed no ORM2 mRNA increase in the heat stress group compared to the control. Therefore, it may be predicted that the augmented ORM2 level in the brain tissues of the heat stress group may not be autocrine in nature that arose from astrocytes. Conversely, the ORM2 possibly reached the brain tissues predominantly secreted from the hepatocytes in the liver, and the increased abundance in the brain was probably due to its increased production in the liver and affected the neuronal tissues via an endocrine route following a leaky blood–brain barrier, though the above pathway may be hypothetical at this point.

While this study delved into the functional aspects of heat stress pathophysiology, it primarily adopted an observational approach, relying on transcriptomic analyses, microbial sequencing, bioinformatic mining, and prediction models. The research, centered on mechanistic validations and pathological evaluations, is confined to identified genes and pathways. Systemic-level and multifactorial measurements including thorough mechanistic insights of the liver-brain mediators are beyond the study's scope. The authors propose subsequent deep investigations into ORM2 mechanisms, connecting the liver-brain axis in heat stress, using tissue-specific knockout models to confirm ORM2's source and eliminate astrocytic ORM2 interference in neuronal etiologies. In conclusion, the study was foundational in employing high-throughput sequencing, explored organ-specific heat stress pathologies, the possible involvement of the gut-liver-brain axis, and molecular interplays, especially in aged mice. The research identified mediators of gut-liver-brain communication, offering implications for developing prognostic and therapeutic markers, crucial for alleviating heat stress pathophysiology, particularly in the aged population.

## Methods

The temperature and humidity-controlled rodent chamber (Model No# RIS26SD) was obtained from Powers Scientific Inc. (Doylestown, PA, USA). Primary antibodies against Cluster of Differentiation 68 (CD68), Translocator Protein (TSPO)/ Peripheral Benzodiazepine Receptor (PBR), and secondary antibodies conjugated with horseradish peroxidase (HRP) were sourced from Abcam (Cambridge, MA, USA). The primary antibody against Orosomucoid 2 (ORM2) was purchased from Proteintech Group Inc (Rosemont, IL, USA). Primary antibodies against IL-1β, IL-6, Brain Derived Neurotrophic Factor (BDNF) were obtained from Santacruz Biotechnology (Dallas, TX, USA). Primary antibodies against Cluster of Differentiation (CD31) and Claudin 5 were acquired from Novus Biologicals, LLC (Centennial, CO, USA) and Sigma-Aldrich (St. Louis, MO, USA) respectively. Species-specific biotinylated secondary antibodies and streptavidin-HRP were obtained from Vector Laboratories (Vectastain Elite ABC kit, Burlingame, CA, USA). Fluorescence-conjugated (Alexa Flour) secondary antibodies and ProLong Gold antifade mounting media with DAPI were purchased from Thermofisher Scientific (Grand Island, NY, USA). The IHC-Tek was acquired from IHCWORLD (Catalog# IW-1000). The brain and liver tissue sections were paraffin-embedded by AML Laboratories (St. Augustine, FL, USA). Unless otherwise specified, all other chemicals used in the study were purchased from Sigma-Aldrich (St. Louis, MO, USA).

### RNA isolation

Samples of frozen tissue were cut and weighed to obtain ≤ 25 mg. The tissue was placed into a 1.7 mL microfuge tube with 500uL Trizol (Life Technologies) and kept on ice. The tissue was homogenized with a disperser (IKA) with three cycles of 20 s of dispersing (speed 6- liver; speed 3- brain) followed by 10 s of rest. The tube was kept in an ice water bath during the homogenization. After the homogenization, the tube was centrifuged for 12,000×*g* for 1 min. The supernatant was removed to a new 1.7 mL microfuge tube without disturbing cell debris that may have been pelleted. The remainder of the protocol followed the instructions for the Zymo Research Direct-zol RNA miniprep kit with some minor edits. An equal volume of ethanol (90–100%) was added to the supernatant and briefly vortexed. The mixture was transferred to the Zymo Spin IICR column in a collection tube and centrifuged for 30 s at 12,000×*g*. The flow-through was discarded and 400uL of Direct-zol RNA Wash Buffer was added to the column. The column was centrifuged for 30 s at 12,000×*g*. A mixture of DNase digestion buffer (75uL) and DNase I (5uL) was added to the column and incubated at room temperature for 15 min. After the DNase I incubation, 400uL of Direct-zol RNA PreWash was added to the column, followed by centrifugation for 30 s at 12,000×*g*. The flow-through was discarded and the PreWash step was repeated. The flow through was discarded and 700uL of Direct-zol RNA Wash Buffer was added to the column and centrifuged for 2 min at 12,000×*g*. The spin column was removed to a fresh collection tube and centrifuged again for 1 min at 12,000×*g*. To elute the RNA, 30uL of DNase/RNase-free water was added directly to the column and incubated for 5 min at room temperature. The RNA was eluted by centrifugation for 1 min at 16,000×*g* into a new 1.7 mL lo-bind RNase-free microtube. The RNA was stored at − 80 °C.

### RNA sequencing

Total RNA was monitored for quality control using the Agilent Bioanalyzer Nano RNA chip and Nanodrop absorbance ratios for 260/280 nm and 260/230 nm. Library construction was performed according to the Illumina TruSeq mRNA stranded protocol. The input quantity for total RNA was ~ 550 ng and mRNA was enriched using oligo dT magnetic beads. The enriched mRNA was chemically fragmented for three minutes. First-strand synthesis used random primers and reverse transcriptase to make cDNA. After second-strand synthesis, the ds cDNA was cleaned using AMPure XP beads, and the cDNA was end-repaired and then the 3’ ends were adenylated. Illumina unique dual indexed adapters were ligated on the ends and the adapter-ligated fragments were enriched by nine cycles of PCR. The resulting libraries were validated by qPCR and sized by Agilent Bioanalyzer DNA high-sensitivity chip. The concentrations for the libraries were normalized and multiplexed together. The multiplexed libraries were sequenced using paired-end 100 cycles chemistry on the NovaSeq 6000. We targeted 20 M reads per sample.

### RNA-Seq data analysis

Sequencing reads were analyzed for quality control using FASTQC (v. 0.11.2), then trimmed using Trimmomatic (v.0.32) with Illumina TruSeq adapter sequences, PHRED quality score 15, and minimum length 20 bases. The trimmed reads were aligned to the reference genome with transcriptome annotation and post-processed using Tophat2 (v.2.0.12), Bowtie2 (v.2.2.3), and Samtools (v.0.1.19). Reads from the same samples were combined. The expression level was quantified both with FPKM (Fragment per kilobase per million mapped reads) using Cufflinks (v. 2.1.1) and with raw counts using HTSeq (v.0.6.1p1.). Differential analysis was performed using DESeq (v.1.18.0).

### RNA sequence analysis

The quality of the sequencing was first assessed using the *fastQC* tool (v0.11.9) on the paired-end reads. Raw reads were then illumina adapter and quality trimmed and filtered by a length of 20 bases using *trimmomatic* (v0.39). Trimmed reads were aligned to indexed mouse reference genome *mm10* using short read aligner *hisat2* (v2.2.1) and gene expression levels were quantified using *featureCount* from *Subread* (v2.0.1) for all annotated genes and samples. Differential gene expression analysis was performed using R package *DESeq2*(v1.22.2) implementing a negative binomial model of the gene count with an FDR (false discovery rate) cut-off of 0.05. PCA plots and heatmaps were generated using *DESeq2*(v1.22.2).

### Pathway and network analysis

The differentially expressed gene lists (DEG) were imported into the *Ingenuity Pathway Analysis* tool (*IPA*: v094302991) using default parameters with a log fold change cutoff of less than − 1.0 and greater than 1.0 for pathway enrichment analysis. Statistically significant canonical pathways were identified and filtered for pathways of interest to create a customized chart. Of the 25 networks generated by IPA, those of interest were selected and exported. *Cytoscape* (v3.10.0) was used to generate protein–protein interaction networks. The DEG lists were filtered for downregulated and upregulated genes using log fold change cutoff of less than − 1.0 and greater than 1.0 and *p* value less than 0.05 and imported into Cytoscape. The *STRING* protein database (v11.5) generated networks based on the gene list. The generated networks were filtered for the largest subnetwork. The final network was generated, and edges were colored by combining log fold change and adjusted p values.

### Animal model

The study used pathogen-free, adult, male, 24 months old C57BL/6 J wild-type (WT) mice obtained from the Jackson Laboratories (Bar Harbor, ME, USA). The mean body weight of the animals used was 36.93 ± 1.69 g at day 0 of the experiment. The mice were housed in a temperature-controlled room with a 12-h light/dark cycle and had ad libitum access to food and water. All experimental procedures were conducted following the guidelines outlined in the NIH Guide for the Humane Care and Use of Laboratory Animals and ARRIVE GUIDELINES. The study protocol was approved by the institutional review board at the University of South Carolina (Animal Protocol Number: 2488-101501-051220; approval date: 5th October 2021). The animals were handled in compliance with local IACUC (Institutional Animal Care and Use Committee) standards. After the completion of the experimental treatments, all mice were euthanized.

### Analysis of gut microbiome

At the conclusion of the experimental timeline, fecal pellets from the control and heat stress mouse groups were subjected to DNA isolation and purification using the ZymoBIOMICS Miniprep kit. Following this, DNA libraries were prepared, and QC was performed using the Qubit dsDNA HS assay from ThermoFisher (Waltham, MA, USA). Whole metagenome sequencing (WMS) was performed on the Illumina NovaseqX high-throughput sequencer, generating 12 million reads per sample conducted by CosmosID (Rockville, MD, USA). The unassembled sequencing reads for each sample were then analyzed using the vendor-optimized bioinformatics platform and pipelines. The bar graph was generated using the ratio value from the observed relative abundance of the bacterial phyla Firmicutes and Bacteroidetes from respective mouse groups.

### Experimental model used

To study the underlying pathological changes in exposure to heat stress, an aging mouse model was used. Aged C57BL6/J (24 months old) mice were exposed to heat stress at 40 ± 0.5 °C with a relative humidity of 60% ± 5% for three hours/day for 15 days mimicking a periodic heat wave exposure. The total number of animals in each group (n = 3) was determined based on statistical power calculations to ensure adequate statistical analysis. The mice were randomly allocated to their respective cages following a randomization procedure. At 24 months and 16 days of age, all mice were euthanized, and serum and liver tissues were collected for further analysis. Brain and liver tissues were fixed in 10% neutral buffered formalin after euthanization to prepare them for sectioning and subsequent processing.

### Immunohistochemistry

The brain and liver tissue sections were deparaffinized according to a standard laboratory protocol, utilizing a solution and steamer from IHC-World (Woodstock, MD, USA) for antigen epitope retrieval. To inhibit endogenous peroxidase activity, a 3% H2O2 solution was applied for 20 min. Subsequently, serum blocking was carried out using 5% goat serum for 1 h. Overnight incubation at 4 °C with primary antibodies (ORM2, CD68, α-SMA, IL-1β, IL-6, TSPO, BDNF), appropriately diluted in a blocking buffer as per the manufacturer’s recommended dilutions, were conducted in a humidified chamber. For detection, species-specific biotinylated secondary antibodies and streptavidin-conjugated with horseradish peroxidase were employed, following standard protocols from the manufacturer. 3,3-diaminobenzidine (DAB) from Sigma-Aldrich (St. Louis, MO, USA) was utilized as a chromogenic substrate to visualize immunoreactivity. Counterstaining with Mayer's hematoxylin from Sigma-Aldrich (St. Louis, MO, USA) was performed. Throughout the procedure, tissue sections were washed with 1X PBS-T (PBS + 0.05% Tween 20) between steps to eliminate any unbound reagents. Finally, the sections were mounted in Aqua Mount from Lerner Laboratories (Stamford, CT, USA). Image acquisition was carried out using an Olympus BX63 microscope (Olympus, Center Valley, PA, USA), and morphometry analysis was performed using Cellsens Software V2.2 from Olympus (Center Valley, PA, USA).

### Immunofluorescence

The formalin-fixed, paraffin-embedded brain tissue sections were deparaffinized following standard instructions. Epitope retrieval of the deparaffinized tissue sections was performed using an epitope retrieval solution and a steamer from IHC World, following the manufacturer's protocol. Primary antibodies for CD31 and Claudin 5 were used at the manufacturer’s recommended dilutions and incubated overnight at 4 °C. Species-specific anti-IgG secondary antibodies conjugated with Alexa Fluor 633 or 488 from Invitrogen (Waltham, MA, USA), USA) were used for detection. The tissue sections were mounted using ProLong Gold antifade mounting media with DAPI from Thermofisher Scientific (Grand Island, NY, USA) which helps preserve fluorescence and provides nuclear staining with DAPI. Images of the tissue sections were captured under 60X magnification using an Olympus BX63 microscope (Olympus, Center Valley, PA, USA). Morphometry analysis was performed using Cellsens Software V2.2 (Olympus, Center Valley, PA, USA).

### ELISA

Serum levels of IL-1β and IL-6 were estimated in the serum samples collected from the mice groups by using the commercially available mouse IL-1β (Cat# KE10003) and IL-6 (Cat# KE10007) ELISA kits, purchased from ProteinTech Group Inc (Rosemont, IL, USA).

### Statistical analyses

Results from transcriptomics were analyzed through a test of significance using the False Discovery Rate (FDR) method providing P_FDR_ Values (P_FDR_ < 0.05). The other results were presented as mean ± standard error of the mean (S.E.M.). Statistical analyses were performed using an unpaired t-test, and significance was determined by the Bonferroni Dunn post hoc correction method using GraphPad Software V10.1.0 (San Diego, CA, USA). A *p* value of less than 0.05 (p < 0.05) was considered statistically significant.

## Data Availability

The RNA-seq data generated in this study has been submitted to the NCBI Gene Expression Omnibus (https://www.ncbi.nlm.nih.gov/geo/). The accession number is GSE252887.

## References

[1] Perkins-Kirkpatrick SE, Lewis SC (2020). Increasing trends in regional heatwaves. Nat. Commun..

[2] National Academies, Sciences, and Engineering; Health and Medicine Division; Board on Population Health and Public Health Practice; Committee on the Respiratory Health Effects of Airborne Hazards Exposures in the Southwest Asia Theater of Military Operations. *Respiratory Health Effects of Airborne Hazards Exposures in the Southwest Asia Theater of Military Operations*. 10.17226/25837 (National Academies Press, Washington, DC, US, 2020). 33030852

[3] Lugo-Amador, N. M., Rothenhaus, T. & Moyer, P. Heat-related illness. *Emerg. Med. Clin. North Am.* **22**, 315–327, viii (2004). 10.1016/j.emc.2004.01.004 10.1016/j.emc.2004.01.004 15163570

[4] Ebi KL (2021). Hot weather and heat extremes: health risks. Lancet.

[5] QuickStats: Percentage Distribution of Heat-Related Deaths,* by Age Group - National Vital Statistics System, United States, 2018–2020. *MMWR Morb. Mortal Wkly. Rep.* **71**, 808 (2022). 10.15585/mmwr.mm7124a6 10.15585/mmwr.mm7124a6 35709072

[6] Malmquist A, Hjerpe M, Glaas E, Karlsson H, Lassi T (2022). Elderly People's perceptions of heat stress and adaptation to heat: An interview study. Int. J. Environ. Res. Public Health.

[7] Knowlton K (2007). Projecting heat-related mortality impacts under a changing climate in the New York City region. Am. J. Public Health.

[8] Hayhoe K (2004). Emissions pathways, climate change, and impacts on California. Proc. Natl. Acad. Sci. USA.

[9] McMichael AJ, Lindgren E (2011). Climate change: present and future risks to health, and necessary responses. J. Intern. Med..

[10] Sharma HS, Cervos-Navarro J (1990). Brain oedema and cellular changes induced by acute heat stress in young rats. Acta Neurochir. Suppl. (Wien).

[11] Sharma HS, Cervos-Navarro J, Dey PK (1991). Acute heat exposure causes cellular alteration in cerebral cortex of young rats. Neuroreport.

[12] Sharma HS (1992). Age-related pathophysiology of the blood-brain barrier in heat stress. Prog. Brain Res..

[13] Lee W, Moon M, Kim HG, Lee TH, Oh MS (2015). Heat stress-induced memory impairment is associated with neuroinflammation in mice. J. Neuroinflamm..

[14] Watt MJ, Miotto PM, De Nardo W, Montgomery MK (2019). The liver as an endocrine organ-linking NAFLD and insulin resistance. Endocr. Rev..

[15] Lin H, Decuypere E, Buyse J (2006). Acute heat stress induces oxidative stress in broiler chickens. Comp. Biochem. Physiol. A Mol. Integr. Physiol..

[16] Ma B (2022). Chronic heat stress causes liver damage via endoplasmic reticulum stress-induced apoptosis in broilers. Poult. Sci..

[17] Tang LP (2022). Heat stress in broilers of liver injury effects of heat stress on oxidative stress and autophagy in liver of broilers. Poult. Sci..

[18] Supplitt S, Karpinski P, Sasiadek M, Laczmanska I (2021). Current achievements and applications of transcriptomics in personalized cancer medicine. Int. J. Mol. Sci..

[19] Irmady K (2023). Blood transcriptomic signatures associated with molecular changes in the brain and clinical outcomes in Parkinson's disease. Nat. Commun..

[20] Munji RN (2019). Profiling the mouse brain endothelial transcriptome in health and disease models reveals a core blood-brain barrier dysfunction module. Nat. Neurosci..

[21] Costa V, Aprile M, Esposito R, Ciccodicola A (2013). RNA-Seq and human complex diseases: recent accomplishments and future perspectives. Eur. J. Hum. Genet..

[22] Srikanth K (2019). Cardiac and skeletal muscle transcriptome response to heat stress in kenyan chicken ecotypes adapted to low and high altitudes reveal differences in thermal tolerance and stress response. Front Genet.

[23] Lu Z (2019). Transcriptomic analysis provides novel insights into heat stress responses in sheep. Animals (Basel).

[24] Li G (2023). Transcriptomic regulations of heat stress response in the liver of lactating dairy cows. BMC Genomics.

[25] Koch F (2019). Heat stress directly impairs gut integrity and recruits distinct immune cell populations into the bovine intestine. Proc. Natl. Acad. Sci. USA.

[26] Jovic K (2017). Temporal dynamics of gene expression in heat-stressed Caenorhabditis elegans. PLoS ONE.

[27] Sammad A, Luo H, Hu L, Zhu H, Wang Y (2022). Transcriptome reveals granulosa cells coping through redox, inflammatory and metabolic mechanisms under acute heat stress. Cells.

[28] Bouchama A (2017). A model of exposure to extreme environmental heat uncovers the human transcriptome to heat stress. Sci. Rep..

[29] Breschi A, Gingeras TR, Guigo R (2017). Comparative transcriptomics in human and mouse. Nat. Rev. Genet..

[30] Li L (2023). Orm2 deficiency aggravates high-fat diet-induced obesity through gut microbial dysbiosis and intestinal inflammation. Mol. Nutr. Food Res..

[31] Xia B (2022). Heat stress-induced mucosal barrier dysfunction is potentially associated with gut microbiota dysbiosis in pigs. Anim. Nutr..

[32] Hu C (2022). Heat stress-induced dysbiosis of porcine colon microbiota plays a role in intestinal damage: A fecal microbiota profile. Front. Vet. Sci..

[33] Magne F (2020). The firmicutes/bacteroidetes ratio: A relevant marker of gut dysbiosis in obese patients?. Nutrients.

[34] Jasirwan COM, Muradi A, Hasan I, Simadibrata M, Rinaldi I (2021). Correlation of gut Firmicutes/Bacteroidetes ratio with fibrosis and steatosis stratified by body mass index in patients with non-alcoholic fatty liver disease. Biosci. Microbiota Food Health.

[35] Grigor’eva IN (2020). Gallstone disease, obesity and the firmicutes/bacteroidetes ratio as a possible biomarker of gut dysbiosis. J. Pers. Med..

[36] Li L (2023). Mitigation of non-alcoholic steatohepatitis via recombinant Orosomucoid 2, an acute phase protein modulating the Erk1/2-PPARgamma-Cd36 pathway. Cell Rep..

[37] Zhu HZ (2020). Downregulation of orosomucoid 2 acts as a prognostic factor associated with cancer-promoting pathways in liver cancer. World J. Gastroenterol..

[38] Jo M (2017). Astrocytic orosomucoid-2 modulates microglial activation and neuroinflammation. J. Neurosci..

[39] Wan JJ (2019). Role of acute-phase protein ORM in a mice model of ischemic stroke. J Cell Physiol.

[40] Cruz-Rivera YE (2018). A selection of important genes and their correlated behavior in Alzheimer's disease. J. Alzheimers Dis..

[41] Srour M (2016). Gain-of-function mutations in RARB cause intellectual disability with progressive motor impairment. Hum. Mutat..

[42] Gao XL (2020). High-mobility group nucleosomal binding domain 2 protects against microcephaly by maintaining global chromatin accessibility during corticogenesis. J. Biol. Chem..

[43] Sun X, Lin Y (2016). Npas4: Linking neuronal activity to memory. Trends Neurosci..

[44] Fu J, Guo O, Zhen Z, Zhen J (2020). Essential functions of the transcription factor npas4 in neural circuit development, plasticity, and diseases. Front Neurosci..

[45] Kanekiyo T, Bu G (2014). The low-density lipoprotein receptor-related protein 1 and amyloid-beta clearance in Alzheimer's disease. Front Aging Neurosci.

[46] Storck SE, Kurtyka M, Pietrzik CU (2021). Brain endothelial LRP1 maintains blood-brain barrier integrity. Fluids Barriers CNS.

[47] Nikolakopoulou AM (2021). Endothelial LRP1 protects against neurodegeneration by blocking cyclophilin A. J. Exp. Med..

[48] Michetti F (2023). The S100B protein: A multifaceted pathogenic factor more than a biomarker. Int. J. Mol. Sci..

[49] Sen J, Belli A (2007). S100B in neuropathologic states: The CRP of the brain?. J. Neurosci. Res..

[50] Milovanovic J (2020). Interleukin-17 in chronic inflammatory neurological diseases. Front Immunol..

[51] Reverchon F (2020). Hippocampal interleukin-33 mediates neuroinflammation-induced cognitive impairments. J. Neuroinflamm..

[52] Rao X (2022). Dual roles of interleukin-33 in cognitive function by regulating central nervous system inflammation. J. Transl. Med..

[53] Andreadou M (2023). IL-12 sensing in neurons induces neuroprotective CNS tissue adaptation and attenuates neuroinflammation in mice. Nat. Neurosci..

[54] Yu T, Flores-Solis D, Eastep GN, Becker S, Zweckstetter M (2023). Phosphatidylserine-dependent structure of synaptogyrin remodels the synaptic vesicle membrane. Nat. Struct. Mol. Biol..

[55] Janz R (1999). Essential roles in synaptic plasticity for synaptogyrin I and synaptophysin I. Neuron.

[56] Wang ZM (2020). SerpinA3N deficiency deteriorates impairments of learning and memory in mice following hippocampal stab injury. Cell Death Discov..

[57] Kim BM, Abdelfattah AM, Vasan R, Fuchs BC, Choi MY (2018). Hepatic stellate cells secrete Ccl5 to induce hepatocyte steatosis. Sci. Rep..

[58] Mohs A (2017). Functional role of CCL5/RANTES for HCC progression during chronic liver disease. J. Hepatol..

[59] Kern P (2021). Creld2 function during unfolded protein response is essential for liver metabolism homeostasis. FASEB J..

[60] Ma W, Huang G, Wang Z, Wang L, Gao Q (2023). IRF7: role and regulation in immunity and autoimmunity. Front. Immunol..

[61] Zangara MT, Johnston I, Johnson EE, McDonald C (2021). Mediators of metabolism: An unconventional role for NOD1 and NOD2. Int. J. Mol. Sci..

[62] Omaru N, Watanabe T, Kamata K, Minaga K, Kudo M (2022). Activation of NOD1 and NOD2 in the development of liver injury and cancer. Front Immunol..

[63] Sun H, Feng J, Tang L (2020). Function of TREM1 and TREM2 in liver-related diseases. Cells.

[64] Monin L, Gaffen SL (2018). Interleukin 17 family cytokines: Signaling mechanisms, biological activities, and therapeutic implications. Cold Spring Harb. Perspect. Biol..

[65] Liu T (2020). The IL-23/IL-17 pathway in inflammatory skin diseases: From bench to bedside. Front Immunol.

[66] He S (2021). LRG1 is an adipokine that mediates obesity-induced hepatosteatosis and insulin resistance. J. Clin. Invest..

[67] Xu L (2023). LRG1 inhibits hepatic macrophage activation by enhancing TGF-beta1 signaling to alleviate MAFLD in mice. Nan Fang Yi Ke Da Xue Xue Bao.

[68] Ghaleb AM, Yang VW (2017). Kruppel-like factor 4 (KLF4): What we currently know. Gene.

[69] Yan M (2023). Gut liver brain axis in diseases: the implications for therapeutic interventions. Signal Transduct Target Ther..

[70] Chatterjee S, More M (2023). Cyanobacterial harmful algal bloom toxin microcystin and increased vibrio occurrence as climate-change-induced biological co-stressors: exposure and disease outcomes via their interaction with gut-liver-brain axis. Toxins (Basel).

[71] Zhou B, Luo Y, Ji N, Hu C, Lu Y (2022). Orosomucoid 2 maintains hepatic lipid homeostasis through suppression of de novo lipogenesis. Nat. Metab..

[72] Elpek GO (2021). Orosomucoid in liver diseases. World J. Gastroenterol..

[73] Castell JV (1989). Interleukin-6 is the major regulator of acute phase protein synthesis in adult human hepatocytes. FEBS Lett..

[74] Ridker PM, Rane M (2021). Interleukin-6 signaling and anti-interleukin-6 therapeutics in cardiovascular disease. Circ. Res..

